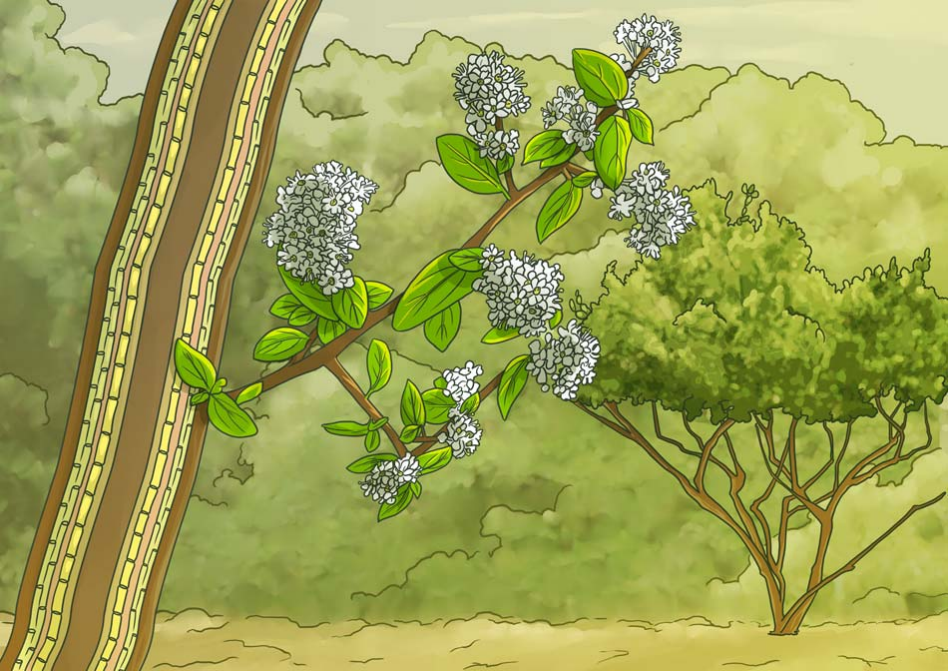# When less is more: failure to adapt to local conditions sometimes boosts resilience

**DOI:** 10.1093/conphys/coab055

**Published:** 2021-07-14

**Authors:** Alison Haynes

**Affiliations:** Centre for Sustainable Ecosystem Solutions, University of Wollongong, New South Wales 2522, Australia

When it comes to survival on land, adapting to local conditions is generally an advantage. But, what if a plant that has not fine-tuned to its environment turns out to be better suited to survive climate change?

Could this be?

Aaron Ramirez and his University of California colleauges think so, especially upon investigating island plant populations to identify potential climate refugia. As temperatures rise and droughts increase with global climate change, the team’s findings offer hope for plant conservation.

Identifying climate refugia is particularly important for plants because plants cannot move. To date, most studies have used spatial analysis combined with climate predictions to identify a refuge for a species. Islands are special because their climates are often moderated by maritime conditions and therefore tend to be wetter than the mainland. But what if, over time, island plants adapt to the extra moisture? If that is the case, then these island populations will fare no better than mainland populations when the entire region dries.

This is why, when identifying a potential climate refuge for a plant, it is crucial to consider the plant’s physiology as well as local climate factors. That is exactly what Ramirez’s team set out to do. They compared two plant populations in California—one population on the Channel Islands, which are relatively wet, and the other on the (drier) mainland.

For over three years, the scientists focused on 20 shrub species and measured a range of drought-related responses, including ‘hydraulic failure’, which is when the water transport tissues (i.e. the xylem) can no longer function, which causes the plant to collapse.

The team’s findings were surprising. Island plants had no differences in their hydraulic traits than mainland plants. In other words, although the island is wetter, island plants had not fine-tuned their traits to suit the gentler climate but instead retained a wide hydraulic safety margin and could still tolerate the drier conditions of the mainland. Overall, island plant populations, then, can tolerate a greater change in conditions than their mainland counterparts and would therefore be more resilient to drought.

Why haven’t the island plants not adapted their hydration traits to their local environment? The scientists offer three possibilities. First, as seedlings, the island plants might need the mainland level of drought tolerance. Second, trait thresholds could be set during extreme events (e.g. drought) and retained, even if conditions are normally benign. However, it is the third hypothesis that the researchers strongly support. There is not a sufficient cost or disadvantage—think natural selection—to maintaining these hydration traits, and so hydration traits are retained, even if they are barely needed.

What does this mean? This study highlights that, when identifying climate refugia, it is important to consider an organism’s physiology, for example their sensitivity to certain conditions, as well as the environmental conditions of the habitat. Ramirez’s study reminds us that navigating climate change is complex, but showcases a useful tool for conserving plants—the members of the ecosystem that cannot simply move if conditions become unfavourable. Sometimes maintaining a wider window of opportunity—not adapting fully—has its benefits.

When it comes to evolution, sometimes, less ismore.

Illustration by Erin Walsh; Email: ewalsh.sci@gmail.com